# Book Reviews

**Published:** 1913-09

**Authors:** 


### BOOK REVIEWS.

---

Sexual Impotence. Wm. J. Robinson of New York, published by Critic & Guide Co., 12 Mt. Morris Pk., W., N. Y; 422 pages; $3.00.

Dr. Robinson discusses the numerous phases of this subject, in both sexes, clearly and in detail. He tells no lies to conform to moral, social and religious ideals and, consequently, those who differ with him in beliefs or in pretensions may censure him as immoral. In some of these points there is opportunity for difference of opinion but, on the whole, we think that Dr. Robinson has expressed what the majority of physicians believe, though not necessarily the opinion most frequently published.

The scope of the work is rather inadequately expressed by the title, as pretty nearly every conceivable sexual abnormality, physical or psychic, is at least alluded to. While the author necessarily deals with subjective symptoms and impressions, there is not the tedious and disgusting display of the patient's mental evacuations which are so conspicuous in some works along comparable lines and for which we have never been able to see any more excuse than for giving the patient's introspection full play in typhoid, hepatic sclerosis and other diseases. If we were to select any one feature of this work for special mention, it would be the uniform common sense of the author—not implying on one hand that there is any lack of scientific thoroughness nor, on the other, that the author's judgment is always the last word in debated topics.

---

Ionic Medication. Lewis Jones, M. D., London; P. Blakiston's Son & Co., Philadelphia; 151 pages; $1.50.

After discussing very lucidly the theory of ionization and giving tables of electro-chemic equivalents, etc., the author passes to the consideration of apparatus and methods. As the subject is practically limited to galvanism, this part is quite brief. Diseases and drugs are then considered seriatim, the author limiting himself to the conditions to which the method is practically applicable and to the means which have been found of actual service. Cautions and contra indications are plainly stated. Perhaps the best recommendation of the work is its brevity, for the reason that the very clear presentation of principles and facts has not been obscured by a mass of unnecessary details and that the therapeutic portion of the book is limited to items apparently well established.

---

Genito-Urinary Diagnosis and Therapy. By Dr. Ernst Portner of Berlin, translated and edited by Bransford Lewis, M. D., B. S., St. Louis. Published by the C. V. Mosby Co., St. Louis; 221 pages; 43 illustrations; $2.50.

This is a brief but full consideration of the venereal diseases and other diseases and lesions of the genito-urinary organs of the male and female. The apothecary's system is used exclusively in the formulæ. The author, or editor, is to be complimented on having, for the most part, avoided such objectionable words as Bartholinitis, Vaginitis, etc. An appendix by Dr. A. Sophian of Kansas City, deals with the serologic test for and the vaccine treatment of gonococcic disease. The work is of the highest practical value.

Report of the Commissioner of Education (P. P. Claxton) Department of the Interior, 1912. Government Printing Office, Washington; two volumes.

The first volume deals with general discussions of educational systems in this and foreign countries; the second is almost entirely statistic. Approximately 20 per cent. of the whole population is enrolled in grammar schools or high schools, and from 68 to 78 per cent. of the population between 5 and 18 years of age is thus enrolled in different sections. Illiteracy shows some curious and significant variations. For the whole country, 7.7 per cent of the population over 10 years of age is illiterate. 30.4 per cent. of negroes, 12.7 per cent. of foreign-born and only 3.7 per cent. of natives born of native parents are illiterate. But the native born of foreign or mixed parentage have the lowest illiteracy of all—1.1 per cent. Such figures seem susceptible of only two interpretations: either the 12.7 per cent. illiteracy of the foreign born is illiteracy in regard to a language still unfamiliar or, if accurate for any language, it is the illiteracy of lack of opportunity and not of appreciation of the value of education.

Approximately half of the population from 15 to 17 inclusive, is at school, so that it may be concluded that nearly half of the rising generation have a high school education. For the years 18-20, the proportion drops sharply to 15 per cent., but there are over a third of a million students who have reached their majority. The time, direct expense and abstraction of so much of the population from productive labor as well as the maintenance of a correspondingly large proportion of the adult population as teachers, while of true economy, represents a burden many times greater than that of the army and navy and, doubtless, has some influence on the increasing stress of living. Perhaps, looking toward the future, it would be well to apply seriously, to education, the joke about the man who offered his sword to his country and who was refused because what the country most needed was muskets.

---

The *New Zealand Medical Journal* of June, 1913, reviews six American books, two English books and three small works by British colonists.

Acknowledgment is made of the receipt of the Annual Calendar of the Faculty of Medicine and of the Department of Dentistry and a separate fasciculus of the latter. These are for the session of 1913-14, being the 82d of the Department of Medicine and the 10th of the Department of Dentistry.

A REFERENCE HANDBOOK OF THE MEDICAL SCIENCES. Embracing the entire range of Scientific and Practical Medicine and Allied Science. By various writers. Third edition, completely revised and rewritten. Edited by Thomas Lathrop Stedman, A. M., M. D. Complete in eight imperial quarto volumes. Volume II. Wm. Wood & Co., New York.

The second volume maintains the high standard of the first and the policy of the present edition in presenting a relatively greater number of short articles than in either of the previous editions, is more apparent in the second volume. We find that the first edition, published about 1888-90, is still not only interesting but valuable for many purposes aside from historic reference. The present edition is not only up-to-date but perfected by experience. Among the contributors we note the following names of men in our special field: McGuire, Mann, Park and Benedict of Buffalo, Kerr of Ithaca.

---

PRACTICAL MEDICINE SERIES. Vol. 3, Eye, Ear, Nose and Throat; Vol. 4, Gynaecology. The Year Book Publishers, Chicago. Vol. 3, 370 pages, illustrated, $3.50; Vol. 4, 230 pages, $1.35. Price for the annual series of ten volumes, $10.

This, series, which we have reviewed favorably for several years, is under the general editorial charge of Charles L. Mix, A. M., M. D., of Chicago, who was formerly associated with the late Dr. Gustavus P. Head in the same capacity. Vol 3 is under the special editorial control of Dr. Head, Casey A. Wood and Albert H. Andrews. Vol. 4 is edited by Drs. Emilius C. Dudley and Herbert M. Stowe, all of Chicago. Being a review of current literature, the series does lend itself well to detailed review. All things considered, it combines brevity, wise selection and thoroughness of detail, in an ideal way.

---

GOUT, ITS AETIOLOGY, PATHOLOGY AND TREATMENT, by James Lindsay, M. D., M. R. C. P., London, formerly Honorable Pathologist and Resident Medical Officer of the Royal Mineral Water Hospital of Bath. Published by the Joint Committee of Henry Frowde and Hodder & Stoughton, Oxford Press, London, represented for America by the Oxford University Press, 35 W. 32 St., N. Y. City; 212 pages, $1.50.

The thoroughness and impartiality with which the author has collated his data, his large personal experience of a practical nature but freedom from hobbies, render it extremely difficult to review this book. There is scarcely any theory of its nature seriously proposed and accepted by many physicians at any time, which does not contain a germ of truth, and none which by itself, explains the pathogeny of all cases. Necessarily, in some instances, one has to deal with conflicting evidence. Consequently, the book should be read in full and the tendency to follow any tangent of aetiology or treatment should be resisted. The fact that the author has based his book on both literary study and personal experience and that he is qualified to control chemic and pathologic data by clinical results will prove disappointing to such readers as ask a simple, general, easily remembered theory and schedule of therapeutics. But the same qualifications will increase the value and interest of the book to the thorough student who wishes to have precise information and guidance toward a successful therapy for the individual case. Finally, just to be inconsistent, we wish to throw out a suggestion that the conspicuous types and frequency of gout in England and in some parts of France, may have a very simple geologic explanation—the richness of the soil in chalk, at least in certain portions of southern England. This may also explain certain apparent inconsistencies with regard to race, habits of eating and drinking, exercise, heredity, etc. And it may also explain the efficacy, in certain cases, of apparently opposite methods of chemic treatment, the non-association of typic gout and urinary calculus and various other points. If this hypothesis is anything more than the first impression of a conspicuous geologic difference, it may perhaps be tenable that the extreme types of gout are due to the coincidence of fundamental metabolic disturbances with opportunities for impregnation with lime.

---

SKIN DISEASES IN GENERAL PRACTICE, by Haldin Davis, M. B., B. Ch., B. A., F. R. C. S., M. R. C. P., London. Published as above; 340 pages, illustrated, $3.75.

After a brief introductory chapter, the author discusses skin diseases due to pyogenic bacteria, erysipelas, impetigo, boils, furuncles, carbuncles, sycosis, then eczema, whose definition and classification is still unsatisfactory and syphilis. He then adopts a simple classification, especially serviceable to the general practitioner, according as the eruption is wide spread, or confined to various parts as the face, limbs, pudenda, etc.

